# New perspectives on assessment and understanding of the patient with cranial bone defect: a morphometric and cerebral radiodensity assessment

**DOI:** 10.3389/fsurg.2024.1329019

**Published:** 2024-02-06

**Authors:** Arthur Maynart Pereira Oliveira, Almir Ferreira De Andrade, Leonardo Zumerkorn Pipek, Corrado Iaccarino, Andres M. Rubiano, Robson Luis Amorim, Manoel Jacobsen Teixeira, Wellingson Silva Paiva

**Affiliations:** ^1^ Department of Neurosurgery, Hospital das Clínicas HCFMUSP, Faculdade de Medicina, Universidade de Sao Paulo, São Paulo, Brazil; ^2^ Department of Neurology, Hospital das Clínicas HCFMUSP, Faculdade de Medicina, Universidade de Sao Paulo, São Paulo, Brazil; ^3^ Department of Biomedical, Metabolic and Neural Sciences, University of Modena and Reggio Emilia, Modena, Italy; ^4^ Department of Neurosurgery, Universidad de Bogotá Jorge Tadeo Lozano, Bogotá, Colombia; ^5^ Centre for Neuroscience in Education, University of Cambridge, Cambridge, United Kingdom

**Keywords:** cranioplasty, decompressive craniectomy, trephined syndrome, head CT, morphometric changes, skull defects

## Abstract

**Background:**

Skull defects after decompressive craniectomy (DC) cause physiological changes in brain function and patients can have neurologic symptoms after the surgery. The objective of this study is to evaluate whether there are morphometric changes in the cortical surface and radiodensity of brain tissue in patients undergoing cranioplasty and whether those variables are correlated with neurological prognosis.

**Methods:**

This is a prospective cohort with 30 patients who were submitted to cranioplasty and followed for 6 months. Patients underwent simple head CT before and after cranioplasty for morphometric and cerebral radiodensity assessment. A complete neurological exam with Mini-Mental State Examination (MMSE), modified Rankin Scale, and the Barthel Index was performed to assess neurological prognosis.

**Results:**

There was an improvement in all symptoms of the syndrome of the trephined, specifically for headache (*p* = 0.004) and intolerance changing head position (*p* = 0.016). Muscle strength contralateral to bone defect side also improved (*p* = 0.02). Midline shift of intracranial structures decreased after surgery (*p* = 0.004). The Anterior Distance Difference (ADif) and Posterior Distance Difference (PDif) were used to assess morphometric changes and varied significantly after surgery. PDif was weakly correlated with MMSE (*p* = 0.03; *r* = −0.4) and Barthel index (*p* = 0.035; *r* = −0.39). The ratio between the radiodensities of gray matter and white matter (GWR) was used to assess cerebral radiodensity and was also correlated with MMSE (*p* = 0.041; *r* = −0.37).

**Conclusion:**

Morphological anatomy and radiodensity of the cerebral cortex can be used as a tool to assess neurological prognosis after DC.

## Introduction

Skull defect after decompressive craniectomy (DC) is related not only to aesthetic and socioeconomic implications but also to physiological changes in brain function (1–5). Patients' complaints usually begin a few weeks after DC. The most common symptoms are dizziness, discomfort in the incision, intolerance to positional changes, and pain at the incision site (6, 7). Moreover, soft tissue depression in the brain can cause more serious complications such as Syndrome of the trephined (ST) or Sinking Skin Flap Syndrome (SSFP). The conditions include neurological deficits that may be evident such as loss of contralateral muscle strength, cognitive changes, and cortical irritating symptoms such as seizures (8, 9).

There are several doubts about the pathophysiology involved in neurological complaints that precede cranioplasty. Possible causes include atmospheric pressure (10), changes in dynamics of cerebrospinal fluid circulation and brain venous drainage (11), changes in the metabolism and cerebral hemodynamics (5, 12–15).

Most studies that try to assess those changes in patients use perfusion computed tomography (PCT). One of the findings in PCT is increased cerebral blood flow, usually up to seven days after cranioplasty (13, 14). However, this increase in a tissue that is being compressed and suffering could mean merely impairment of cerebral self-regulation. This alteration could cause reactive hyperemia with increased cerebral blood flow (CBF), as has already been observed after DC, and which is not always related to an improvement in cerebral hemodynamics (16). In addition, Sarubbo et al. (17) showed that after cranioplasty, PCT may show an initial increase in CBF (hyperemia) followed by a drop of approximately 50% of these values after three months of surgery, raising further doubts about the value of CBF assessment in patients with cranial bone defect.

Several aspects related to the benefits of cranioplasty in these patients still need a better explanation. Recently, some studies have shown more objectively the changes related to the influence of atmospheric pressure and how changes in the dynamics of cerebrospinal fluid (CSF) circulation could occur in these patients (15, 18, 19). However, the explanation of how these changes trigger brain dysfunction is not yet established.

Some studies show that the amount of water in the brain, which can be measured by computed tomography (CT) through the evaluation of the Hounsfield units (Hu) of the white matter (SB) and gray matter (SC), could be a prognostic factor for patients who have suffered cardiorespiratory arrest (20) and for victims of traumatic brain injury (21). These measures could be useful in an attempt to assess non-invasively and indirectly the amount of water in the neuronal interstitium of the cerebral cortex.

The objective of this study is to evaluate whether there are morphometric changes in the cortical surface and radiodensity of brain tissue in patients undergoing cranioplasty and whether there would be any capacity for CT scan of the skull to predict neurological prognosis in these cases.

## Methods

### Study design

This was a single-center prospective cohort. Patients were followed up for 6 months. The study was approved by the ethics board of the research committee (in accordance with the Helsinki Declaration, revised in 1983), and all patients signed the informed consent form. The study is reported based on the STROBE statement (22).

### Participants

Patients who were previously subjected to DC based on recommendations from clinical guidelines for the management of severe TBI and stroke (23, 24) were asked to undergo cranioplasty. No selection regarding the reasons for DC (traumatic brain injury or cerebrovascular disease) was performed. A prior diagnosis of ST was not necessary for inclusion. The exclusion criteria were as follows: patients younger than 18 years old, loss of follow-up before 6 months, or development of deep infection after cranioplasty requiring removal of the bone or prosthesis.

### Cranioplasty

The cranioplasty was performed in a standard manner with autologous bone or with methyl methacrylate polymer when the bone was not available. The surgery was performed under general anesthesia with the patient in the supine position with cephalic lateralization contralateral to bone defect. Surgery preparation included trichotomy and rigorous asepsis with chlorhexidine. The previous incision was reopened with careful separation of the cutaneous flap from the dura mater. When possible, the temporal muscle was also isolated and separated from the dura mater, but in most cases, there was already atrophy and very intense adhesion.

Autologous bone was replaced with a nylon 2.0 fixation and, when it was not possible, prostheses molded during the procedure made from methyl methacrylate were used. In all cases, 4–8 fixation were made from the dura mater to the bone or prosthesis with a prolene suture to reduce dead space and try to bring it as close as possible to its original position. In all cases, a drain under the galea was used with a vacuum for 24 h. All patients were followed up, discharged on the fifth day, and stitches removed on the fourteenth postoperative day.

### Evaluation of the morphological anatomy and radiodensity of the cerebral cortex

All patients underwent simple head CT before and after 15–30 days of cranioplasty. A 64-channel multidetector CT scanner (Philips Medical Systems World Headquarters, Best, the Netherlands) was used for imaging acquiring. The method used for calculating the horizontal surface of the bone defect was as described by Sarov M & investigators et al. (7). The following formula was used: π/4 × A × B (“A” is the longest anteroposterior distance between the internal edges of the bone and “B” the longest upper to lower distance from the edge of the craniotomy).

All exams were evaluated by two radiologists experienced in the area of neuroimaging and blinded to the patient's clinical condition. The presence of cortical gyri and sulci were assessed using the entire sequence of images. All other analyses were made at the level of the foramen of Monro, both before and after the surgery.

Initially, we evaluate the shift of intracranial structures along the midline, known as the Midline Shift (MLS). This is quantified in millimeters (mm) and is measured as the distance from the midline to the pellucid septum.

Following this, we proceed to analyze the cortical mantle's restructuring, which may include compression or potential expansion of the cortex surface post-surgery. This analysis is conducted in two specific regions - the frontal region and the temporal lobe.

For the frontal region, we first locate a point two cm posterior to the internal bone board along the midline. From this point, a line is drawn perpendicular to the surface of the frontal cortex. This measurement is termed the Anterior Distance (AD).

In the case of the temporal lobe, we identify a point six cm posterior to the internal bone board along the midline. A line is then drawn perpendicular from this point to the temporal cortex's surface. This measurement is referred to as the Posterior Distance (PD).

To accommodate the possible anatomical variations between individuals, we perform these measurements on both sides of the brain and derive the absolute difference. For instance, the Anterior Distance Difference (ADif) is computed by subtracting the anterior distance on the normal side (ADN) from that on the cranioplasty side (ADC).

Subsequently, we track the change in these distances pre and post-surgery. For example, the Anterior Variation is calculated as the difference between the ADif before surgery and the ADif after surgery.

A similar process is followed for the measurements pertaining to the temporal lobe, allowing us to calculate the Posterior Distance Difference (PDif) and the Posterior Variation.

**Figure 1:**
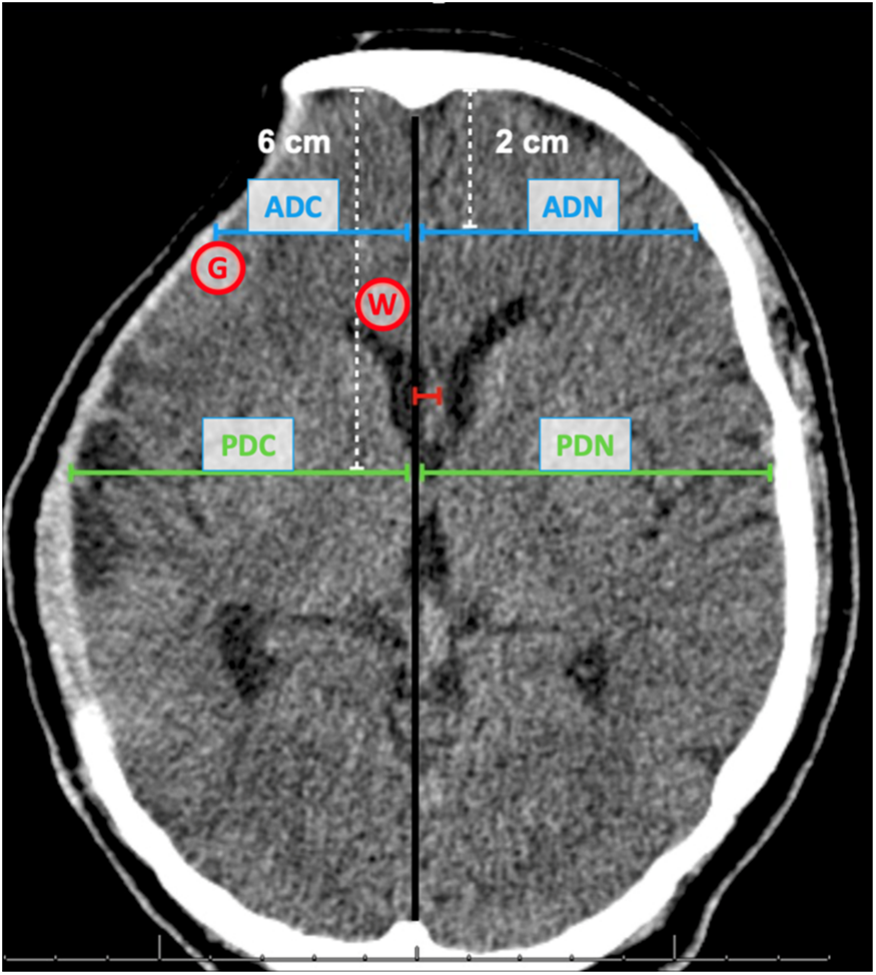
Frontal and temporal measurements. Compression of the frontal region (anterior distance - blue line) and temporal region (posterior distance - green line). The midline shift distance is represented in red. Anterior distance cranioplasty side (ADC); Anterior distance normal side (ADN); Posterior distance cranioplasty side (PDC); Posterior distance normal side (PDN); Area for Graw Matter radiodensity (G); Area for White Matter radiodensity (W).

After the morphometric analysis, we performed the radiodensity assessment measured in Hounsfield Units (HU) in two pre-determined regions of interest (ROI) before surgery and after 15–30 days of cranioplasty. In order for our evaluation to be standardized in all cases, we selected an ROI of 3 cm^2^ immediately after the location where the ADC line met the frontal cortex, exactly at the limit of the cerebral cortex surface to assess the gray matter (GM) radiodensity. To analyze the white matter (WM) radiodensity, we selected the region of the knee of the corpus callosum, immediately anterior to the lateral ventricle, in the same tomographic section at the level of the foramen of Monro, and homolateral to the bone defect (Figure 1). Unlike the previously described methodology that used the region of the base nuclei to measure the density of the gray matter (20), we opted to use the radiodensity of the gray matter in the cerebral cortex, since our goal is to try to evaluate the changes provided by compression of the cerebral cortex in patients with bone defect. In addition, for each patient, we also calculate the ratio between the radiodensities of GM and WM (GWR) in an attempt to better interpret any loss of GM-WM differentiation.

### Assessment of neurological prognosis

We evaluated symptoms related to the presence of Syndrome of the trephined (ST) and Sinking Skin Flap Syndrome (SSFS) and other neurological prognosis scales using data from before cranioplasty and six months after. A questionnaire for common complaints of dizziness, tinnitus, headache, or seizures was used. Symptoms related to changes in the position of the cephalic segment, such as discomfort in the incision and intolerance to movement were obtained. Data from a complete neurological examination was also obtained: muscle strength, deep tendon reflexes, superficial and deep sensitivity, static and dynamic balance, and cranial nerves. For prognosis assessment, scales were applied with an evaluation of the Mini-Mental State Examination (MMSE), modified Ranking Scale (mRS), and the Barthel Index.

### Statistical analysis

We performed the Kolmogorov–Smirnov test to assess the normality of the data. Non-parametric tests were used for the analysis. Data were expressed as mean and standard deviation, median and interquartile range, or absolute frequency and percentage. The difference in means before and after surgery was tested with the Student's *t*-test for paired data (for data from neuroimaging). To assess the scales of MMSE, Barthel, and mRS, the Wilcoxon test was used, and its data are expressed as median and Interquartile Interval Q1–Q3. For muscle strength assessment before and after surgery, the Wilcoxon test was also used, while for qualitative SoT variables, the McNemar test was used. All tests were two-tailed and the level of significance was set at 0.05. The analyzes were performed using the Statistical Package for Social Sciences (SPSS) software version 13.0 for Windows (Chicago, IL, United States).

## Results

### Baseline population characteristics

The study enrolled 30 patients (20 men and 10 women) who were submitted to cranioplasty and followed for 6 months. The mean age was 41 ± 12.61 years. Half of the patients underwent DC as a result of skull trauma and the other half related to stroke. Regarding the size of the bone defect, we observed that the mean surface area of the craniectomy was 105.02 cm^2^ ± 26.17 cm^2^. The average anterior/posterior and inferior/superior diameter was 12.96 cm ± 2.17 cm and 10.18 cm ± 1.26 cm, respectively (Table 1).

**Table 1:** Baseline characteristics.

Age	41.0 ± 12,6
Male	20 (66%)
Etiology	
TBI patients	15 (50%)
Stroke patients	15 (50%)
Left side cranioplasty	17 (56%)
Craniectomy distance cm	
A/P	12.9 ± 2.1
U/l	10.1 ± 1.2
Craniectomy surface area cm^2^	105.0 ± 26.1
Cranioplasty technique	
Methyl methacrylate	16 (53%)
Autologous	14 (47%)
Waiting time to cranioplasty in months	15.2 ± 17.6
More than 3 months for surgery	26 (87%)

TBI, traumatic brain injury; A/P, anterior/posterior; U/l, upper/lower.

Data reported as mean ± SD or number (%).

### Neurologic outcomes

Regarding the signs and symptoms of ST, we observed that 3 patients (10%) had seizures in the initial evaluation with only 1 patient (3.3%) maintaining crises after cranioplasty (*p* = 0.5). Likewise, 4 patients (13.3%) had dizziness before cranioplasty and only 1 (3.3%) afterwards (*p* = 0.25). Movement intolerance showed significant improvement (*p* = 0.016) with a reduction of symptoms in 87.5% of the 8 patients who had this symptom before surgery. Regarding the improvement in symptoms of discomfort in the incision and tinnitus, 7 (23.3%) and 3 (10%) patients who presented these symptoms, respectively, none of them complained after the cranioplasty (Table 2).

**Table 2:** Clinical assessment before and after cranioplasty.

Syndrome of the trephined symptoms	Cranioplasty		*p*-value
	Before	After (6 months)	
Headache	11 (36.7%)	2 (6.7%)	**0** **.** **004** *
Convulsion	3 (10%)	1 (3.3%)	0.500
Intolerance changing head position	8 (26.7%)	1 (3.3%)	**0**.016*
Incision pain	7 (23.3%)	0 (0%)	**0.033** * **^a^**
Tinnitus	3 (10%)	0 (0%)	0.317**^a^**
Dizziness	4 (13.3)	1 (3.3%)	0.250

Data reported as number (%).

* *p*-value <0.05.

^a^ McNemar test with Yates correction.

During the evaluation of muscle strength contralateral to bone defect side, we observed a significant improvement (*p* = 0.02) of this parameter. No case presented worsening of strength, 20 (66.6%) remained with the same degree of strength after cranioplasty and 10 (33.3%) showed improvement. In the MMSE evaluation, we obtained a median of 23 (22–26 IQR) points before surgery and 25 (23–27 IQR) points after cranioplasty, showing a significant increase (*p* < 0.001). We also observed a significant improvement in the scores obtained in the evaluation of mRS with a reduction in its median from 2.5 (1–4.0 IQR) to 2.0 (1–3.35 IQR) after the correction of bone defect (*p* = 0.003). Despite not having changed its median of 80 points, the Barthel Index shows a significant deviation (*p* = 0.002) increasing its IQR when comparing the assessments before (60–90 IQR) and after cranioplasty (63.75–100 IQR) (Table 3).

**Table 3:** Clinical outcome assessment.

		Before Surgery	After Surgery (6 months)	*p*-value
Muscle strength	0	2 (6.7%)	2 (6.7%)	**0** **.** 02*
	1	1 (3.3%)	1 (3.3%)	
	2	2 (6.7%)	1 (3.3%)	
	3	8 (26.7%)	5 (16.7%)	
	4	8 (27.7%)	7 (23.3%)	
	5	9 (30%)	14 (46.7%)	
mRS	mean ± SD	2.57 ± 1.47	2.17 ± 1.51	**0**.003*
	median (IQR)	2.5 (1–4.0)	2.0 (1–3.35)	
MMSE	mean ± SD	23.14 ± 3.99	25.00 ± 3.08	< 0.001*
	median (IQR)	23 (22–26)	25 (23–27)	
Barthel index	mean ± SD	73.50 ± 20.09	79.33 ± 19.37	**0**.002*
	median (IQR)	80 (60–90)	80 (63.75–100)	

mRS, modified rankin scale; MMSE, mini-mental state examination.

Bold values are statistically significant (*p* < 0.05).

### Imaging assessment

On the side without bone defect, all 30 patients had cortical sulci visible on simple head CT before and after surgery. On the operated side, we observed that 10 (33.3%) patients had visible sulci before the surgery. Of the 20 (66.6%) patients without visible sulci in the preoperative period, there was an improvement in 16 (80%, *p* < 0.001). Cortical sulci were present in 86.6% of patients after cranioplasty.

Regarding the deviation of the structures from the midline, we observed a significant reduction from 2.43 ± 2.55 mm (mean and SD) before cranioplasty, to 1.16 ± 1.93 mm (mean and SD) after cranioplasty (*p* = 0.004) (Table 4).

**Table 4:** Cortical morphometric and radiodensity assessment before and after cranioplasty.

	Before Cranioplasty	After Cranioplasty	*p*-value
Cortical Sulci			
Present	10 (33.3%)	26 (86.6%)	< 0.001*
Effaced	20 (66.6%)	4 (13.3%)	
Midline Shift	2.43 ± 2.55	1.16 ± 1.93	**0**.004*
Morphometric			
ADN	44.5 ±** **3.82	43.97 ±** **3.81	0.363
ADC	35.31 ± 6.62	41.02 ± 4.96	< 0.001*
ADif	9.04 ± 7.04	2.94 ± 4.57	< 0.001*
PDN	59.19 ± 3.94	58.98 ± 3.52	0.434
PDC	54,07 **±** 8.68	56.67 ± 4.57	**0**.045*
PDif	5.12 ± 8.77	2.31 ± 3.92	0.063
Radiodensity - Hounsfield unit			
Frontal - GM	28.7 ± 8.02	30.51 ± 7.94	0.144
Corpus callosum – WM	27.08 ± 3.80	26.11 ± 3.82	0.052
GWR	1.05 ± 0.26	1.18 ±** **0.27	**0**.007*

ADN, anterior distance normal side; ADC, anterior distance cranioplasty side; ADif, anterior distance difference; PDN, posterior distance normal side; PDC, posterior distance cranioplasty side; PDif, posterior distance difference; GM, gray matter; WM, white matter; GWR, GM radiodensity/WM radiodensity.

Bold values are statistically significant (*p* < 0.05).

We were able to observe a significant increase both in the ADC from 35.3 ± 6.62 mm to 41.02 ± 4.96 mm (*p* < 0.001) and in PDC from 54.07 ± 8.68 to 56.67 ± 4.57 mm (*p* = 0.045). In addition, there was a significant reduction in ADif from 9.04 ± 7.04 mm to 2.94 ± 4.57 mm (*p* < 0.001). The same assessment was made for the posterior reference point, which showed a non-significant reduction in PDif from 5.12 ± 8.77 mm to 2.31 ± 3.92 mm (*p* = 0.063). Although we did not observe a significant variation in the PDif, when we calculated the ratio instead of the difference between these two measures, we observed a significant variation both for the previous reference point (ADC before/ADC after, *p* < 0.001) and at the posterior (PDC before/PDC after, *p* = 0.047). To assess whether those two ways to assess the change in the distances were correlated, we applied the Spearman test. The test showed an almost perfect correlation between ratio and difference (*r* = 0.99 and *p* < 0.001).

When assessing brain tissue density, we observed an increase in frontal GM density after surgery from 28.7 ± 8.02 UH to 30.51 ± 7.94 UH while for WM there was a reduction of this value from 27.08 ± 3.80 UH to 26.11 ± 3.82 UH, with no significance for any of the evaluated regions. However, we observed that there was a significant increase in GWR after cranioplasty (*p* = 0.007).

By associating changes in morphometry with the variation in the prognosis before and after surgery, we observed that the improvement in the sulcal effacement, alterations in the deviation of the structures of the midline, and the diameter of the cranioplasty showed no significant correlation with the neurological prognosis. However, we were able to observe that the variation in the GWR before and after cranioplasty showed a weak correlation with the MMSE (*p* = 0.041; *r* = −0.37) and that the Posterior Variation (DifP before - DifP after surgery) also showed a correlation with the MMSE (*p* = 0.03; *r* = −0.40) and the Barthel index (*p* = 0.035; *r* = −0.39). (Table 5) Subgroup analysis for TBI and cerebrovascular disease show similar results (Supplementary Material S2).

**Table 5:** Cortical morphometric and radiodensity correlation with prognosis scales and strength power in the neurological examination.

	mRS	MMSE	Barthel Index	Strength Power
Presence of cortical sulci	0.009*	0.868	0.203	0.314
Midline Shift	0.917 (*r* = 0.02)	0.839 (*r* = 0.04)	0.725 (*r* = 0.06)	0.786 (*r* = 0.05)
C*r*aniectomy Su*r*face	0.712 (*r* = 0.07)	0.696 (*r* = −0.07)	0.579 (*r* = 0.10)	0.714 (*r* = 0.07)
GW*r*	0.727 (*r* = 0.06)	0.041 (*r* = −0.37)*	0.076 (*r* = −0.24)	0.133 (*r* = −0.28)
ADif	0.173 (*r* = 0.25)	0.618 (*r* = 0.95)	0.904 (*r* = 0.23)	0.808 (*r* = −0.04)
PDif	0.084 (*r* = −0.32)	0.03 (*r* = −0.40)*	0.035 (*r* = −0.39)*	0.139 (*r* = −0.27)

ADif, anterior distance difference; PDif, posterior distance difference; GWR, gray matter and *p*-value (correlation value). GWR, Gray matter white mater radiodensities ratio; mRS, modified rankin scale; MMSE, mini-mental state examination.

Bold values are statistically significant (*p* < 0.05).

## Discussion

Several late complications can be observed after DC. One of the main findings is the compression of the cortical surface, associated or not with disorders of the CSF circulation with the formation of hygromas and hydrocephalus. According to Yamaura et al. (8), there are four patterns of deformity/compression of the cortical surface: sinking, flat, full, and bulging. In their work, patients were predominant with a sinking surface, which also showed the highest percentage of neurological improvement after cranioplasty (33.3%). This classification, however, can be questioned since it is subjective and dependent on the examiner's impression during visual inspection. In addition, there is a possibility that the anterior portion of the frontal cortex is sunk while the posterior part is flat or bulged, as was observed in several cases in our cohort, making it hard to properly categorize. The aim of this study was to determine an objective method to assess brain morphometry.

The relationship between the presence of cortical sulci and neurological improvement has been described in the literature with a few case reports, but never in a systematic way (25, 26).

The improvement in cortical sulci appearance is probably due to a reduction in the compression exerted by the atmospheric pressure. There is a restructuring of the cortical surface and improvement of CSF circulation underlying the cranioplasty, allowing better tomographic definition of the cortex-CSF interface. Lilja-Cyron et al. (18) studied this restructuring using ICP measurements with intra-parenchymal monitors implanted after DC up to 8 weeks after discharge from the ICU. The results demonstrated that the absence of the skullcap can make de ICP oscillate 5–10 mmHg between the supine and sitting or standing position. In addition, it showed important changes in brain pulse waves, which explain sulci effacement and impaired movement of intracranial fluids. In our study, 86.6% of patients remain or evolve to a condition of visible sulci on cranial CT, but showed no association with improvement in neurological outcomes.

Another parameter of cranial tomography that can be analyzed in patients with large bone defects is the MLS, which is directly related to the compression exerted by atmospheric pressure and consequent impairment of the dynamics of the CSF circulation. George et al. (27) were one of the first to observe this change in the MLS. In his work, using cerebral angiography, he suggests that the patients who showed the most relevant clinical improvement were those who presented the greatest regression of the MLS after cranioplasty. More recently, Lin et al. (28) observed that patients who had MLS before cranioplasty showed a significant improvement in GCS (*p* = 0.03) but did not progress with significant motor improvement assessed by upper limb (*p* = 0.40) and lower limb (*p* = 0.47) strength. In our study, we observed that there was a significant reduction in the MLS after cranioplasty (*p* = 0.004), however, there was no significant correlation between variation in the MLS and recovery of muscle strength or favorable clinical outcomes.

As the classification for patterns of brain tissue after DC previously reported by Yamaura et al. (8) (sinking, flat, full, and bulging) could vary between observers, we developed two objective measures (ADC and PDC) that could reflect the variation of the cortical surface. The anterior reference point (ADC, ADif) reflects the most anterior frontal deformity and, therefore, could be related to cortical functions of the frontal lobe; the posterior reference point (PDC, PDif), which is located almost at the mean distance from the craniectomy, could infer the deformity of the temporal/parietal lobe and its respective functions.

Several reports in the literature show that patients with a sinking cranial flap tend to show neurological improvement, but without quantifying or correlating this anatomical variation of the cortical surface with neurological improvement (7, 8, 29, 30). In our study, we observed that both ADC and PDC showed a significant increase after DC. This data corroborates with most of the literature that describes variations of the cortical surface, however, this is the first study to describe it objectively. In addition, we observe that there is a correlation between brain morphometry variations with neurological prognosis. PDif was correlated with MMSE (*p* = 0.03; *r* = −0.4) and Barthel index (*p* = 0.035; *r* = −0.39).

Although several studies describe that one of the main hypotheses to explain the cerebral cortex dysfunction related to SSFS is the reduction in cerebral blood flow (1, 4, 9, 13, 14, 17), values from most studies are mostly within or close to normal. Normal CSF values are approximately 50 ml/100 g/min (31). Sarubbo et al. (17) evaluated six patients and showed that the mean CBF on the side with bone defect was 75.6 ± 26.0 ml/100 g/min before cranioplasty, increasing to 89.4 ± 19.2 ml/100 g/min 7 days after surgery and reaching a value close to normal of 46.7 ± 15.1 ml/100 g/min in three months after surgery. Paredes et al. (14) evaluated 49 patients and observed an increase in the mean cerebral blood flow of the defective side from 101.86 ml/100 g/min to 117.17 ml/100 g/min 72 h after the cranioplasty. Consequently, we should question whether the main responsible for the clinical improvement of patients would be the increase in these values, or if there is a possibility of error due to possible cerebrovascular reactivity in these cases.

Certainly, CSF is important in the evolution of patients with bone defect, but perhaps the simple comparison between the values of CSF before and after is not the best way to evaluate these cases. We believe that changes in cortical morphometry with impairment of CSF dynamics (sulci effacement) and compression with deformation of the cortical surface may be more relevant to explain cortical dysfunction in SSFS and two hypotheses could explain these conclusions.

In addition to morphometry variations, white and gray matter radiodensity is another parameter that may provide key information. The GWR is directly related to the content of fluids in the cell interstitium. GWR has already been demonstrated to correlate with neurological prognosis in the evaluation of patients who suffered cardiorespiratory arrest (CRP), mainly due to the great variation in the radiodensity of gray matter in these cases. Choi et al. (20) showed lower mean values of the GWR in the group of patients who had CRP (1.21) compared to the control group (1.32) with *p* < 0.001 and determined a cutoff of 1.22 for GWR with specificity and 100% positive predictive value to predict vegetative state or death. For patients suffering from TBI, a similar assessment was made by Kim et al. (21). The study showed that in a group of 300 patients separated by favorable and unfavorable prognosis, GWR was 1.27 (IQR: 1.23–1.31) and 1.27 (IQR: 1, 20–1.33), respectively. In this way, there was no difference between the groups, however, the individual values for white matter and gray matter radiodensity showed a reduction with a strong correlation with an unfavorable prognosis. Although we did not observe significant variations in the radiodensity of gray matter and white matter alone, there was a significant increase in the values of the GWR from 1.05 ± 0.26 to 1.18 ± 0.27 after surgery (*p* = 0.007) and a correlation of GWR with neurological prognosis (MMSE, *p* = 0.041; *r* = −0.37).

The quantitative assessment of GWR is important in assessing the loss of gray/white matter differentiation, and, for patients with bone defect, it can be considered an assessment tool. In our study, we observed that after cranioplasty there was an increase in the radiodensity of gray matter and a reduction in white matter. Although not significant, it indirectly shows us a possible improvement in fluid dynamics on the cortical surface. Before surgery, there may be a dysfunction in the fluid circulation dynamics in the cellular interstitium related to the malfunction of the cerebral cortex Glymphatic system, which in turn causes the impoundment of liquids and a consequent reduction in the radiodensity of gray matter. After surgery, with progressive improvement of fluid dynamics in the cerebral cortex, there would be less damming of liquids in the interstitium with a consequent increase in the radiodensity of gray matter.

Considering the limited number of patients and clinical variables, further research is needed to better determine the factors that might be involved in long-term neurologic outcomes and create prediction models for the outcomes analyzed. Moreover, due to limited resources, the average waiting time for cranioplasty in our study is superior to the recommend, which can influence the outcomes evaluated in the study. Pathophysiological studies are also needed to confirm the possible explanations for clinical improvement after DC. A study population with patients in more severe conditions is also necessary for generalization of our findings.

## Conclusion

The result of this study confirms the benefits of cranioplasty for patients with cranial bone defect. Morphological anatomy and radiodensity of the cerebral cortex can be used as a tool to assess neurological prognosis after DC. PDif was correlated with MMSE and Barthel index, while GWR was increased after surgery and correlated with MMSE.

## Data Availability

The raw data supporting the conclusions of this article will be made available by the authors, without undue reservation.
