# Supplementary material for: New perspectives on assessment and understanding of the patient with cranial bone defect: a morphometric and cerebral radiodensity assessment
Source: Front Surg. 2024 Feb 6;11:1329019. doi: 10.3389/fsurg.2024.1329019 (PMC10876786; doi:10.3389/fsurg.2024.1329019)
Supplement: Supplementary file 1 [file Datasheet1.docx]

Supplement 1: 2 examples of morphometric measurement from patients in our study


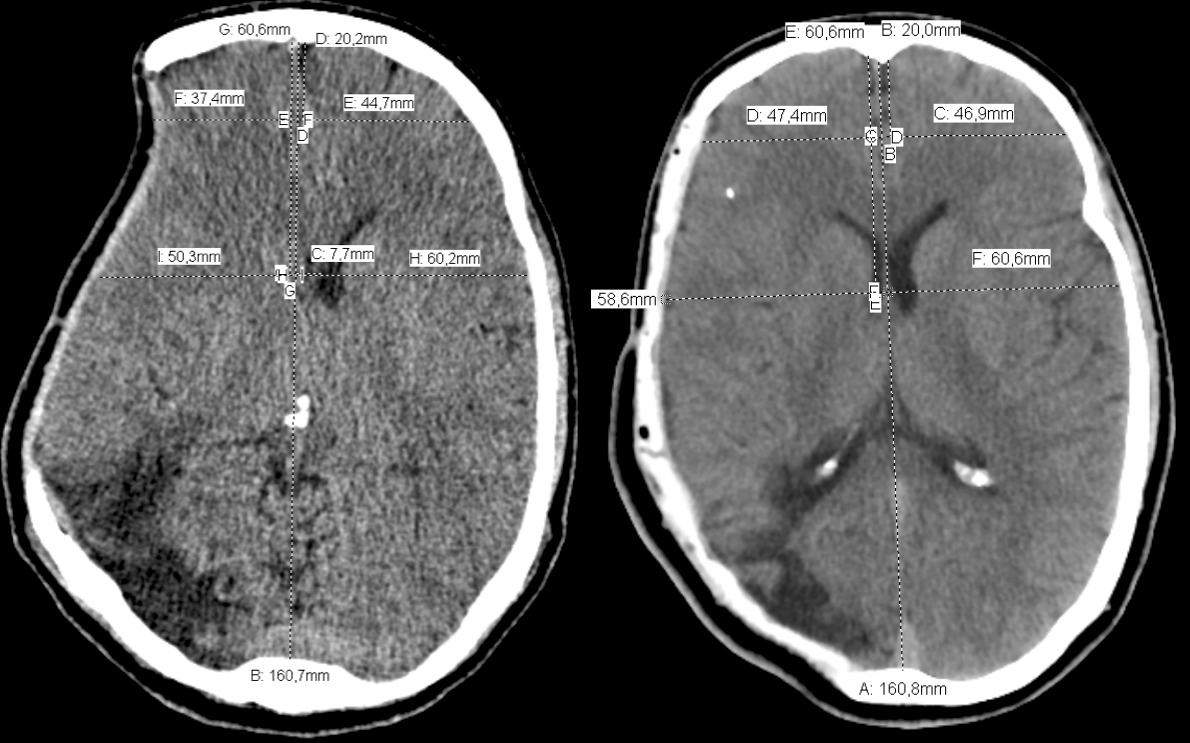


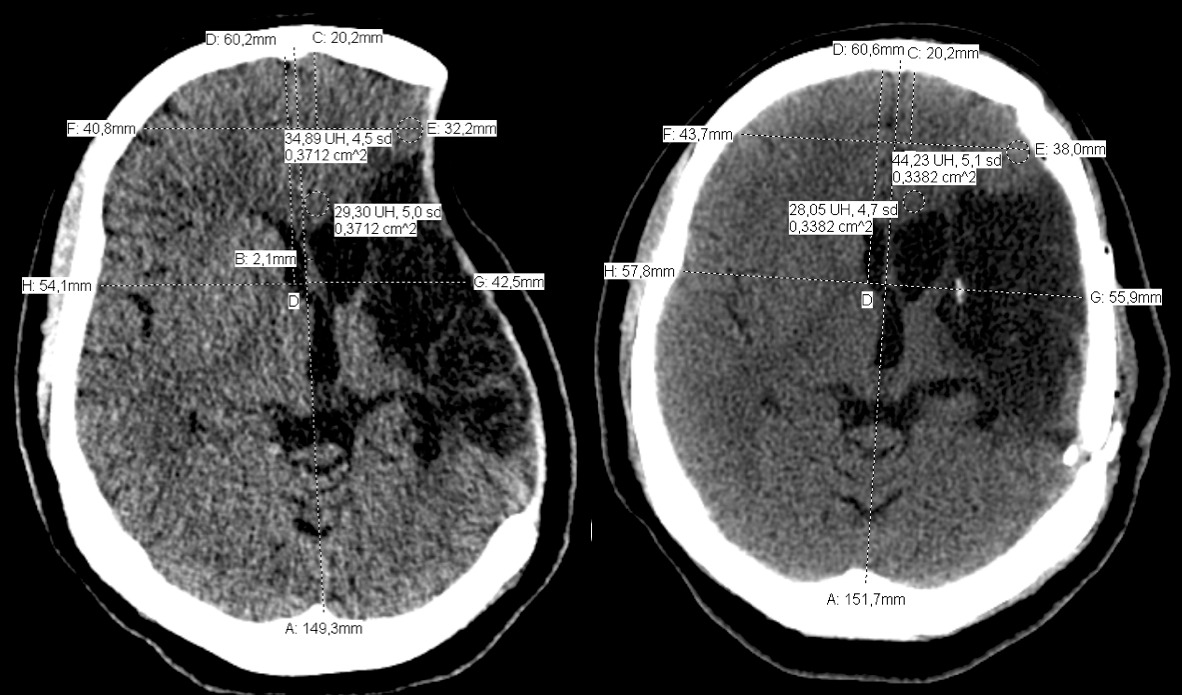


Supplement 2: Subgroup analysis for Cerebrovascular disease and traumatic brain injury.

|  |  | Before surgery | After surgery | p | mRS | MEEM | Barthel Index |
| --- | --- | --- | --- | --- | --- | --- | --- |
| Presence of cortical sulci | TBI | 40% | 80% | 0.031 | - | - | - |
|  |  |  |  |  | - | - | - |
|  | CVD | 26.70% | 93.30% | 0.002 | - | - | - |
|  |  |  |  |  | - | - | - |
| GWR | TBI | 1.06 (0.31) | 1 .13 (0.33) | 0.192 | r= -0.26 | r= 0.57 | r= 0.19 |
|  |  |  |  |  | p= 0.77 | p= 0.083 | p=0.48 |
|  | CVD | 1.05 (0.21) | 1.22 (0.21) | 0.005 | r= 0.143 | r= 0.23 | r= -0.14 |
|  |  |  |  |  | p= 0.61 | p= 0.48 | p=0.61 |
| Midline Shift | TBI | 1 .73 (1.55) | 1.32 (1 .73) | 0.366 | r= -0.81 | r= 0.91 | r= 0.078 |
|  |  |  |  |  | p= 0.77 | p=0.80 | p=0.78 |
|  | CVD | 3.14 (3.16) | 0.99 (2.16) | 0.01 | r=0.13 | r=0.29 | r=0.047 |
|  |  |  |  |  | p=0.62 | p=0.37 | p=0.86 |
| Adif | TBI | 10.44 (6.39) | 4.35(4.6) | 0.6 | r=0.45 | r= -0.21 | r= -0.47 |
|  |  |  |  |  | p=0.092 | p=0.55 | p=0.074 |
|  | CVD | 8.01 (7.17) | 1 .5 (4.92 | 0.002 | r= 0.22 | r= -0.35 | r= -0.09 |
|  |  |  |  |  | p=0.42 | p=0.28 | p=0.72 |
| Pdif | TBI | 4.2 (7.9) | 3.0 (3.8) | 0.6 | r= -0.95 | r= 0.26 | r= -0.11 |
|  |  |  |  |  | p=0.73 | p=0.46 | p=0.69 |
|  | CVD | 6.0 (9.77) | 1.62 (4.05) | 0.095 | r= -0.57 | r= -0.26 | r= 0.54 |
|  |  |  |  |  | p=0.025 | p=0.73 | p=0.036 |

*GWR: Gray matter white mater radiodensities ratio; mRS: modified Rankin Scale; MMSE: Mini-Mental State Examination. TBI: traumatic brain injury; CVD: cerebrovascular disease. ADif*: *Anterior Distance Difference; PDif*: *Posterior Distance Difference*
